# Domain‐Specific Prediction of Clinical Progression in Parkinson's Disease Using the Mosaic Approach

**DOI:** 10.1002/brb3.70289

**Published:** 2025-01-09

**Authors:** Marlene Tahedl, Ulrich Bogdahn, Bernadette Wimmer, Dennis M. Hedderich, Jan S. Kirschke, Claus Zimmer, Benedikt Wiestler

**Affiliations:** ^1^ Department of Neuroradiology, School of Medicine and Health Technical University of Munich Munich Germany; ^2^ Department of Neurology, University Hospital, School of Medicine University of Regensburg Regensburg Germany; ^3^ Department of Neurology, School of Medicine University of Innsbruck Innsbruck Austria

**Keywords:** cortical thickness, machine learning, magnetic resonance imaging, Parkinson's disease, personalized medicine

## Abstract

**Purpose**: Due to the highly individualized clinical manifestation of Parkinson's disease (PD), personalized patient care may require domain‐specific assessment of neurological disability. Evidence from magnetic resonance imaging (MRI) studies has proposed that heterogenous clinical manifestation corresponds to heterogeneous cortical disease burden, suggesting customized, high‐resolution assessment of cortical pathology as a candidate biomarker for domain‐specific assessment.

**Method**: Herein, we investigate the potential of the recently proposed Mosaic Approach (MAP), a normative framework for quantifying individual cortical disease burden with respect to a population‐representative cohort, in predicting domain‐specific clinical progression. Using MRI and clinical data from 135 recently diagnosed PD patients from the Parkinson's Progression Markers Initiative, we first defined an extremity‐specific motor score. We then identified cortical regions corresponding to “extremity functions” and restricted MAP, respectively, and contrasted the explanatory power of the extremity‐specific MAP to unrestricted MAP. As control conditions, domain‐related but less specific general motor function and nondomain‐specific cognitive scores were considered. We also tested the predictive power of the restricted MAP in predicting disease progression over 1 and 3 years using support vector machines. The restricted, extremity‐specific MAP yielded higher explanatory power for extremity‐specific motor function at baseline as opposed to the unrestricted, whole‐brain MAP. On the contrary, for general motor function, the unrestricted, whole‐brain MAP yielded higher power.

**Finding**: No associations were found for cognitive function. The extremity‐specific MAP predicted extremity‐specific motor progression over 1 and 3 years above chance level. The MAP framework allows for domain‐specific prediction of customized PD disease progression, which can inform machine learning, thereby contributing to personalized PD patient care.

## Graphical abstract

Normative assessment of cortical regions corresponding to extremity‐specific motor function (A) at baseline allows to predict associated clinical function (UPDRS extremity subscore) at a 1‐year follow‐up (B). This prediction is specific since unrelated clinical scores (MoCA total score) cannot be predicted (C).

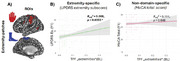

## 1 Introduction

While classically viewed as a disorder predominantly affecting motor function (Kalia and Lang 2015) the clinical presentation of the Parkinson's disease (PD) is multifaceted. Although bradykinesia, rigidity, and tremors are still hallmark diagnostic criteria (Bloem, Okun, and Klein 2021) the role of additional neurological functional domains including nonmotor dysfunctions such as cognitive impairment, sleep disturbance, and psychiatric symptoms are more and more recognized as core features of PD disease manifestation (Greenland et al. 2019). While we are only beginning to understand the significance of the contributions of such nonmotor symptoms to ultimate disease severity, there is growing evidence that even within “classically‐affected” motor domains there is vast heterogeneity in terms of the specifically impaired domain (e.g., bradykinesia and/or rigidity and/or tremor) as well as the speed of progression among the affected patients (He et al. 2023). To enhance personalized medicine, customized diagnostic systems are desirable which may ultimately allow for domain‐specific prediction of symptom manifestation, i.e. individualized disability assessment across neurological functional systems (Mishima et al. 2021). Due to recent advances in computational power, machine learning (ML) offers highly efficient and automatized detection and classification of specific diagnostic categories (Dadu et al. 2022; Nenning and Langs 2022). ML‐based algorithms have been already explored for their utility in predicting specific motor features in PD, such as bradykinesia (Leal et al. 2023). When developing an ML‐based classifier, one crucial step is the selection of features with which to train the algorithm. Due to recent advances in medical image analysis, computationally intensive magnetic resonance imaging (MRI) data can now be easily included in ML feature selection (Shimron and Perlman 2023). The consideration of imaging features for ML might greatly enhance the accuracy of detecting early‐stage changes—potentially even before clinical manifestation—but still informative of future disease unfolding (Domínguez‐Fernández et al. 2023).

One imaging feature with high potential in detecting early‐stage and domain‐specific clinical manifestations is structural cortical markers. Anterograde degeneration of damaged dopaminergic cells in the substantia nigra might only be one mechanism underlying such cortical atrophy (Del Tredici and Braak 2012, 2016; Foffani and Obeso 2018; Horsager et al. 2020; Johansson et al. 2024). Additionally, cortico‐cortical disease propagation is likely contributing to the vast cortical disease manifestation observed in PD (Deng et al. 2021). In this regard, appreciating the highly complex and interconnected nature of the human brain (Rubinov and Sporns 2010), such cortico‐cortical disease propagation can add to explaining the various patterns of clinical manifestations.

Following this line of reasoning, the assessment of cortical disease burden is therefore a candidate biomarker for accurate and customized disability status, prognosis, and prediction in PD. An individualized approach requires assessment of single‐patient deviation from an expected population norm (Bethlehem et al. 2022; Di Biase et al. 2023), ideally for a high‐resolution parcellation of the human cortex to reflect the complex pathogenic pathways as outlined above. The recently proposed Mosaic Approach (MAP) (Tahedl et al. 2021; Tahedl et al. 2024), offers such a system: In brief, MAP uses a parcellation of the human cortex into 1000 roughly equally‐sized patches (Schaefer et al. 2018; Yeo et al. 2011) and calculates, for a given subject, the deviation of each patch with respect to a population‐representative norm from the *CamCAN* MRI repository (Shafto et al. 2014). To correct for physiological confounding effects, an age‐ and sex‐matched subcohort is selected for a given patient (Tahedl et al. 2021), which was shown to maintain variance containing clinical information in various neurodegenerative disorders including the motoneuron disease spectrum (Tahedl et al. 2021; Tahedl et al. 2022b), frontotemporal dementia(McKenna et al. 2022) and multiple sclerosis (MS) (Tahedl et al. 2023; Tahedl et al. 2024). While previous studies have shown that MAP allows for quantification of general clinical disability, we here investigate whether also domain‐specific clinical information can be assessed and even predicted. For that, we restrict MAP to brain regions corresponding to a specific functional domain of interest, using the motor function of the extremities as an example. We expect that this domain‐specific, restricted MAP model yields higher explanatory power for domain‐specific clinical functional assessment as opposed to the whole‐brain, unrestricted MAP approach. As control conditions, we also probe domain‐related but less specific motor functions and nondomain‐specific cognitive functions. While we expect some explanatory power of domain‐specific MAP for the domain‐related but less specific total motor score, the performance of the unrestricted MAP approach should be higher. In contrast, the restricted domain‐specific MAP should yield no explanatory power for the nondomain‐specific cognitive function. Finally, we test the predictive power of the domain‐specific, restricted MAP model as opposed to the unrestricted, whole‐brain model in predicting individual disease progression over 1 and 3 years, employing support vector machines (SVM), which are supervised ML‐based classifiers. We contrast the accuracy of SVMs derived from restricted, domain‐specific, and unrestricted whole‐brain MAP models to predict domain‐specific and domain‐unspecific clinical progressors from nonprogressors over 1 year, expecting the analog tendencies as defined for the cross‐sectional assessment as outlined above. With our study, we aim to investigate the potential of MAP for the prognosis of domain‐specific functional progression, which might ultimately enhance personalized patient follow‐up in PD.

## 2 Methods

### 2.1 Study Participants

Neuroimaging and clinical data were retrieved from the Parkinson's Progression Markers Initiative (PPMI) database (https://www.ppmi‐info.org/about‐ppmi/who‐we‐are/study‐sponsors, accessed on February 15, 2024). PPMI is an international multisite study dedicated to enhancing our understanding of PD etiopathogenesis by collecting and studying data from persons with early PD and as the disease unfolds. Ethical approval and written informed consent were obtained from all participants at the respective scanning sites.

We considered data from untreated PD patients from the initial phase of PPMI, which in total enrolled 423 such patients (https://www.ppmi‐info.org/study‐design/study‐cohorts#pd/). All patients had a clinical diagnosis of PD and a positive dopamine transporter (DAT) SPECT. Further key inclusion criteria of the PPMI initial phase included a minimum age of 18 years, initial PD diagnosis within 7 years, and Hoehn & Yahr stages of 1, 2, or 3 (Hoehn and Yahr 1998) at the time of enrollment.

For the present study, we defined further selection criteria, including a baseline 3 Tesla (3T) T1‐weighted (T1w) MRI scan within 1 year of diagnosis, as well as complete Unified Parkinson's disease rating scale, modified revision sponsored by the Movement Disorder Society (Goetz et al. 2008) (MDS‐UPDRS) and Montreal Cognitive Assessment (MoCA, (Nasreddine et al. 2014)) scores at baseline, at a 1‐ and at a 3‐year follow‐up assessment. These selection criteria resulted in a subcohort of 135 PD patients (53 females) left to analyze for the present study. Details on the demographics and clinical assessments of the study population can be found in Table 1. T1w MRI data at baseline and clinical data at baseline, at a 1‐ and at a 3‐year year follow‐up were considered.

**TABLE 1:** Demographic and clinical data of the study population.

	Baseline (BL)	Year 1 (Y1)	Year 3 (Y3)	Comparison BL vs. Y3 (Wilcoxon signed rank test: *V*, *p*‐value)^*^
Study population [*N* PD patients] (males/females)	135 (82 / 53)	n.a.^**^	n.a.^**^	n.a.
Age [*years*] (M ± SD)	63.46 ± 6.76	n.a.^**^	n.a.^**^	n.a
Handedness (R/L/A)	119/12/4	n.a.^**^	n.a.^**^	n.a.
Diagnosis duration [months] (M ± SD)	4.00 ± 6.50	n.a.^**^	n.a.^**^	n.a.
Symptom duration [months] (M ± SD)	17.00 ± 21.00	n.a.^**^	n.a.^**^	n.a.
“Extremitiy” MDS‐UPDRS III subscore (Mdn ± IQR)	13.00 ± 10.00	15.50 ± 12.00	16.00 ± 14.00	*V* = 2635, *p* = 0.0043^***^
Total MDS‐UPDRS III score (Mdn ± IQR)	20.00 ± 13.00	22.00 ± 17.00	23.00 ± 20.75	*V* = 2261.5, *p* = 0.0012^***^
Total MoCA score (Mdn ± IQR)	28.00 ± 3.00	27.00 ± 4.00	27.00 ± 4.00	*V* = 4088.0, *p* = 0.0009^***^

Abbreviations: A = ambidextrous, IQR = interquartile range, L = left‐handed, M = mean, Mdn = median, MoCA = Montreal Cognitive Assessment, mo = months, *n.a*. = not applicable, P = Parkinson's Progression Markers Initiative, R = right‐handed, SD = standard deviation, MDS‐UPDRS = Unified Parkinson's disease rating scale, modified revision sponsored by the Movement Disorder Society, y = year.

^*^Only for clinical scores (ordinal scales).

^**^PPMI only provides patients’ demographics at baseline (slight dropouts in Y1 and Y3 vs. BL were due to incomplete clinical and/or neuroimaging data).

^***^Significant at *p* < 0.05.

### 2.2 Neuroimaging Data Acquisition

All T1w neuroimaging data were collected on 3T MRI systems, using different manufacturers depending on the scanning site (see also the PPMI MRI Technical Operations Manual v3.0 for more technical details at https://www.ppmi‐info.org) (Marek et al. 2011). Scanners included 3T Siemens (Siemens Medical Solutions), 3T Philips (Healthcare Imaging Systems), and 3T GE (General Electric Healthcare). High‐resolution T1w data were acquired as 3D sequences (either MP‐RAGE or IR‐FSPGR) using the following sequence parameters: 192 sagittal slices (1.0‐1.2 mm slice thickness), voxel size (in‐plane resolution) = 1.0 mm^2^, phase encoding direction = anterior–posterior, matrix size = 256×256 (no interpolation, no zero‐filling), TR/TE/FA were defined by Invicro (*A Konica Minolta Company*, Needham, MA, USA, https://invicro.com) according to the scanner, field of view (FOV) = 256 mm (full FOV, no rectangular), scan time ∼ 7 min. Regarding clinical data, we included baseline and 1‐year follow‐up data from (Goetz et al. 2008) the MDS‐UPDRS and the MoCA (total scores). From the MDS‐UPDRS, we considered two subscores, namely the (1) MDS‐UPDRS III total subscore, which indicates motor function, as well as a (2) composite MDS‐UPDRS III extremity subscore, summing across the following scales: rigidity of four extremities, finger tapping, hand movements, pronation–supination, toe‐tapping, leg agility (MDS‐UPDRS items: 3.3, 3.4, 3.5, 3.6, 3.7, 3.8).

### 2.3 Neuroimaging Data Processing

**FIGURE 1:**
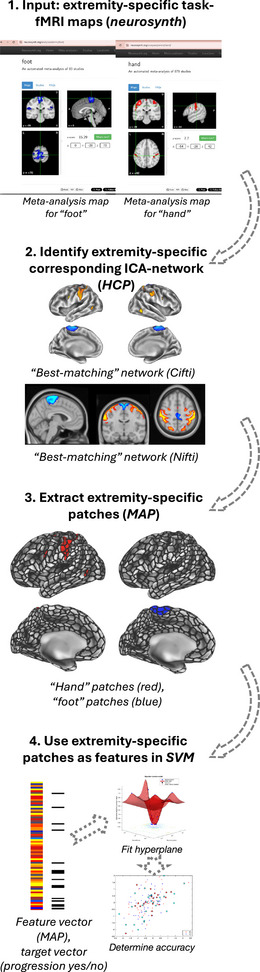
Definition of domain‐specific cortical regions corresponding to “extremity function”. First (panel 1), statistical brain maps corresponding to “hand” and “leg”, to cover both upper and lower extremities, were obtained from the online meta‐analysis tool *NeuroSynth*. We then identified the best‐matching ICA‐based functional brain networks, using high‐resolution fMRI data from the HCP (as recently proposed by (Tahedl and Schwarzbach 2023), panel 2). These maps were then used as masks on the MAP 1000‐parcellation (panel 3), leaving *N* = 79 extremity‐specific patches which were then used as pre‐selected features in an SVM to predict domain‐specific clinical progression (panel 4). HCP = Human Connectome Project, fMRI = functional magnetic resonance imaging, ICA = independent component analysis, MAP = Mosaic Approach, SVM = support vector machine.

T1w anatomical data were preprocessed to obtain corrected surface‐based cortical thickness (CTh) maps using fully‐automated pipelines from the open‐source software FreeSurfer (Fischl 2012) and Ciftify (Dickie et al. 2019). The cortical surface was further subparceled into 500 “patches” or “mosaics” on each hemisphere (the parcellation scheme of the left hemisphere is visualized, e.g., in Figure 1, panel 3) (Buckner et al. 2011; Schaefer et al. 2018). For each patient, normative deviations of each patch were assessed following the recently‐proposed Mosaic Approach (Tahedl et al. 2023; Tahedl et al. 2024): In brief, MAP contrasts individuals observed raw CTh data with that of a large population‐representative cohort from the CamCAN database (*N* > 650). To correct for the physiological effects of age and sex, each patient is compared with an individually selected subgroup from *CamCAN* (same sex, age ± 2 years) (Tahedl et al. 2021). Using that matched reference group, *z*‐scores are calculated for each patch and patient, which are then converted to *p*‐values using exhaustive permutation testing, that is, permutation inference is based on all possible permutations without replacement. (Tahedl 2020). If the *p*‐value of a patch is smaller than 0.05, that patch may be rated as “significantly thin”, that is, indicative of atrophy. The fraction of all “significantly thin” patches, the so‐called “thin‐patch fraction” (TPF), has been shown to mimic clinical disease burden in various neurological disorders, including amyotrophic and primary lateral sclerosis (Tahedl et al. 2021; Tahedl et al. 2022b), frontotemporal dementia (McKenna et al. 2022) and also MS (Tahedl et al. 2023; Tahedl et al. 2024). TPF may be assessed across the whole brain or across a dedicated topographical region of interest. In this study, we investigated the hypothesis that restricting the TPF according to the topographical cortical regions associated with a clinical function of interest may enhance the diagnosis and prognosis of that clinical function.

### 2.4 Definition of “Extremity‐Specific” Patches

In the present study, we investigated whether the domain‐specific restriction of the TPF would enhance the prediction of the corresponding, domain‐specific, clinical function as compared with a nonrestricted TPF and a nondomain‐specific function. Specifically, we evaluated whether restricting the TPF according to the cortical regions associated with “extremity function” at baseline would enhance the domain‐specific clinical prediction, that is, extremity function at baseline, as compared with considering the nonrestricted TPF reflecting the whole brain. As additional control conditions, we evaluated the domain‐specific extremity TPF as well as the nonrestricted whole‐brain TPF at baseline to predict (1) the total MDS‐UPDRS III score, reflecting general motor function, as a less specific but functionally‐related domain‐specific function, and (2) the total MoCA score, as a cognitive and therefore domain‐unspecific function. We defined domain‐specific patches associated with “extremity function” as follows: First, we downloaded statistical brain maps from task‐fMRI meta‐analyses related to “hand” and “leg” function, obtained from the open‐source meta‐analysis tool *NeuroSynth* (https://neurosynth.org/) (Yarkoni et al. 2011) (Figure 1, panel 1). Second, we identified the corresponding resting‐state functional brain networks of those statistical, task‐based brain maps (Figure 1, panel 2). This was achieved following a recently‐proposed pipeline (Tahedl and Schwarzbach 2023), which identifies corresponding functional networks associated with statistical brain maps of interest, by cross‐mapping between task maps and resting‐state ICA functional networks from the large‐scale and high‐quality data repository by the Human Connectome Project (Van Essen et al. 2013). The best‐matching ICA network, corresponding to each “hand” and “motor” function, was then used as a mask on the 1000‐patch parcellation, which resulted in *N* = 79 patches associated with the “extremity motor function” (Figure 1, panel 3).

### 2.5 Statistical Assessment

To identify whether extremity‐specific motor function was better predicted by extremity‐specific cortical patches (CTh, MAP‐standardized) at baseline as compared with considering nonrestricted, whole‐brain patches, we used general linear model statistical assessment: first, we fit linear models, using clinical scores (MDS‐UPDRS III total/MDS‐UPDRS III extremity‐specific/MoCA total, latter as nondomain‐specific control condition) at baseline as dependent variables and TPF from either extremity‐specific or whole‐brain patches at baseline as independent variables. We corrected for confounding effects of age, sex, handedness, and symptom duration. *p*‐values < 0.05 were considered as statistically significant. We compared the models using the adjusted *R^2^* values. All GLM models were computed and visualized within RStudio (RStudio Team 2022).

### 2.6 Patient Definition as “Progressors” and “Non‐Progressors”

We aimed to investigate whether the extremity‐specific restriction of TPF would enhance the domain‐specific classification of PD patients, that is, identifying patients that would deteriorate after 1 and 3 years (“progressors”) from patients that would not deteriorate (“non‐progressors”). We defined three binary classes of PD progressors/nonprogressors, according to changes in clinical/neuropsychological scores across 1 and 3 years (cf. also Figure >3):

1. Domain‐specific progressors: Extremity‐specific motor function progressors (any increase of MDS‐UPDRS III extremity subscores across 1 year/3 years)
2. Domain‐related but less specific progressors: General motor function progressors (any increase of MDS‐UPDRS III scores across 1 year/3 years)
3. Domain‐unrelated progressors: Cognitive progressors (any decrease of MoCA total scores across 1 year/3 years)

### 2.7 Classification

SVM is a family of machine‐learning, supervised classification algorithms, that aims to identify the best hyperplane to separate data from two (binary) classes (Vapnik and Lerner 1963). We evaluated two SVM classifiers for each of the three clinical groups (as defined above), considering information from either

1. the unrestricted TPF, considering all whole‐brain patches (*N* = 1000), or
2. the domain‐specific, reduced number of extremity‐specific patches (*N* = 79)

as input. As input types, that is, features, we used the MAP‐derived *z*‐scores from each patch, indicating individual CTh deviation from the matched reference population (Figure 1, panel 4). We evaluated six final SVM linear classifiers, one for each combination of clinical progression group and input (as listed above). The construction of each SVM model was run using Matlab's *fitcsvm* tool (The Mathworks, Natick, MA, USA) and involved the following steps, based on (Ebadi et al. 2017): First, we split our data set into train/test subset using a ratio of 0.8/0.2. Next, we selected features to reduce the dimensionality of the input data: For the classifier considering all *N* = 1000 patches, we set the number of features‐to‐select to *N* = 79 to enhance comparability to the extremity‐specific classifier, which also used 79 features as input and for which we did not run further features selection, since we already considered that specific subset of patches as pre‐selected. Then, we identified the best hyperparameters using a radial basic function kernel. Finally, we ran a fivefold cross‐validation scheme. The accuracy was then simply calculated as the fraction of correctly classified cases of all considered cases and used as a metric to compare between models. We repeated this process both for the 1 as well as the 3‐year follow‐up.

## 3 Results

### 3.1 Demographic and Clinical Characteristics

In total, data from 135 PD patients (82 males, 53 females) at three timepoints (baseline, 1‐ and 3‐year follow‐ups) were considered for this study. The mean age was 63.46 years with a standard deviation of ±6.76 years. The median diagnosis duration at baseline was 4.00 months (interquartile range 6.50 months). Comparing the 3‐year (Y3) follow‐up versus the baseline assessment, Wilcoxon signed rank tests indicated significantly higher MDS‐UPDRS III extremity subscores at Y3 (*V* = 2635.0, *p* = 0.0043), higher total MDS‐UPDRS III scores at Y3 (*V* = 2261.5, *p* = .0012), and lower MoCA total scores at Y3 (*V* = 4088.0, *p* < 0.001). Further details on demographic and clinical characteristics are provided in Table 1.

### 3.2 Correlations Between Baseline Thin‐Patch Fraction and Baseline Clinical Scores

**FIGURE 2:**
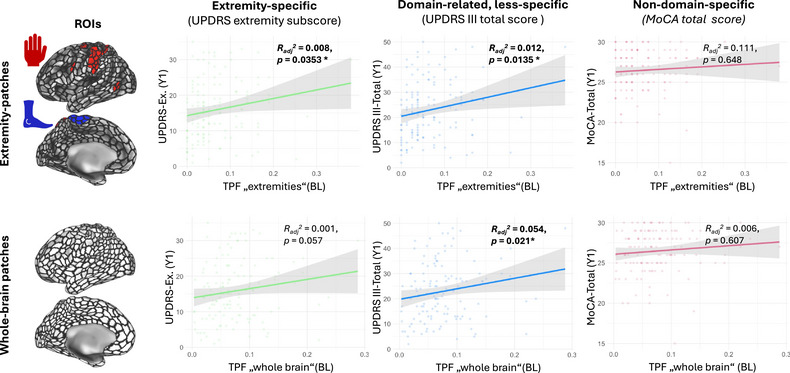
Baseline correlations between TPF and clinical scores. The upper row shows linear best fits of the extremity‐specific restricted TPF, only considering domain‐specific patches topographically associated with “extremity function” (cf. methods section and Figure 1 for further details on the definition of “extremity‐specific patches”). The lower row shows the corresponding correlations for unrestricted whole‐brain TPF. Columns correspond to different clinical functions, namely the extremity‐specific motor function (a subscore from the MDS‐UPDRS III, cf. methods section), domain‐related but less specific general motor function (MDS‐UPDRS III total scores), and domain‐unrelated cognitive function (MoCA total scores). BL = baseline, Ex. = extremity, MoCA = Montreal Cognitive Assessment, ROI = region of interest, TPF = thin‐patch fraction, MDS‐UPDRS = Unified Parkinson's disease rating scale, modified revision sponsored by the Movement Disorder Society.

In our first analysis, we contrasted baseline correlations between the restricted TPF, only including domain‐specific patches topographically associated with “extremity function”, as outlined above, as opposed to the unrestricted whole‐brain TPF. We evaluated these models with regard to (1) extremity‐specific, (2) domain‐related but less specific, and (3) nondomain‐specific clinical scores (Figure 2). The extremity‐specific TPF yielded a significant correlation with the extremity‐specific motor function (*R*_adj_^2^ = 0.045, *p* = 0.035), while the unrestricted whole‐brain TPF did not (*R*_adj_^2^ = 0.021, *p* = 0.208). Furthermore, the extremity‐specific TPF yielded a significant correlation with the total MDS‐UPDRS III score (*R*_adj_^2^ = 0.044, *p* = 0.045), while the unrestricted whole‐brain TPF did not (*R*_adj_^2^ = 0.030, *p* = 0.157). No TPF yielded significant correlations with the domain‐unspecific total MoCA scores (extremity‐specific TPF: *R*_adj_^2^ = −0.012, *p* = 0.911; unrestricted whole‐brain TPF: *R*_adj_^2^ = −0.015, *p* = 0.318).

### 3.3 Patient Definition as “Progressors” and “Non‐Progressors”

**FIGURE 3:**
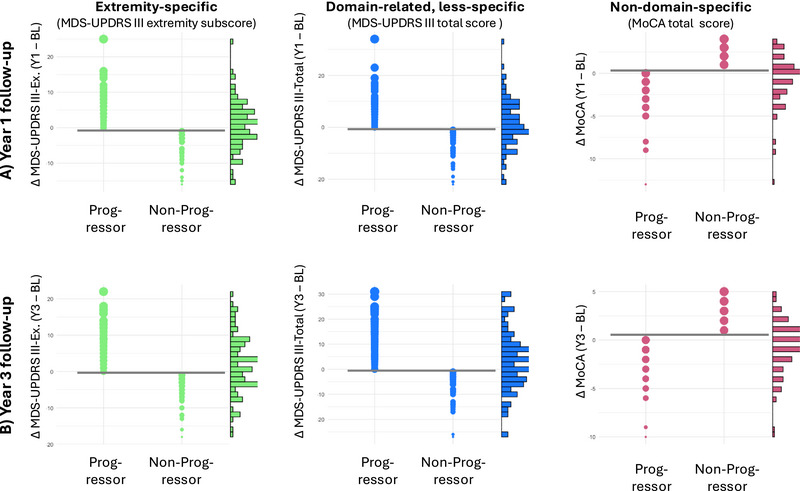
Patient definition as “progressors” and “non‐progressors”. For each analyzed clinical measurement, patients were classified as either “progressors” or “non‐progressors” across 1 year (Y1, A)/3 years (Y3, B). For extremity‐specific motor function and domain‐related but less specific general motor function (both subscores from the MDS‐UPDRS III), progressors were defined as patients with an increased score at the Y1/Y3 follow‐up assessment. For nondomain‐specific cognitive function, progressors were defined as patients with a decreased MoCA total score at Y1/Y3. Each dot indicates the difference (Δ) of the respective clinical score at Y1–BL/Y3‐BL, the histograms to the right are the corresponding distributions. The thick horizontal line in gray marks the zero‐line, that is, the cutoff between progressors and nonprogressors. BL = baseline, Ex. = extremity, MoCA = Montreal Cognitive Assessment, ROI = region of interest, TPF = thin‐patch fraction, MDS‐UPDRS = Unified Parkinson's Disease Rating Scale, modified revision sponsored by the Movement Disorder Society, Y1 / Y3 = 1‐/3‐year (follow‐up assessment).

We classified patients as “progressors” and “non‐progressors” based on the difference scores between the Y1/Y3 follow‐ups and the baseline clinical assessments (Figures 3A,B). For MDS‐UPDRS III total and extremity‐specific subscores, progressors were defined as patients with an increased score at Y1/Y3, and vice versa for nondomain‐specific MoCA scores (note higher MDS‐UPDRS III but lower MoCA scores reflect worse clinical function). For each clinical measure, the group sizes were roughly equally sized (Y1: MDS‐UPDRS III extremity‐specific: *N* = 73 progressors, MDS‐UPDRS III total: *N* = 73 progressors, MoCA: *N* = 87 progressors; Y3: MDS‐UPDRS III extremity‐specific: *N* = 75 progressors, MDS‐UPDRS III total: *N* = 83 progressors, MoCA: *N* = 87 progressors).

### 3.4 Prediction of Clinical Progression Using Baseline Thin‐Patch‐Fraction

**FIGURE 4:**
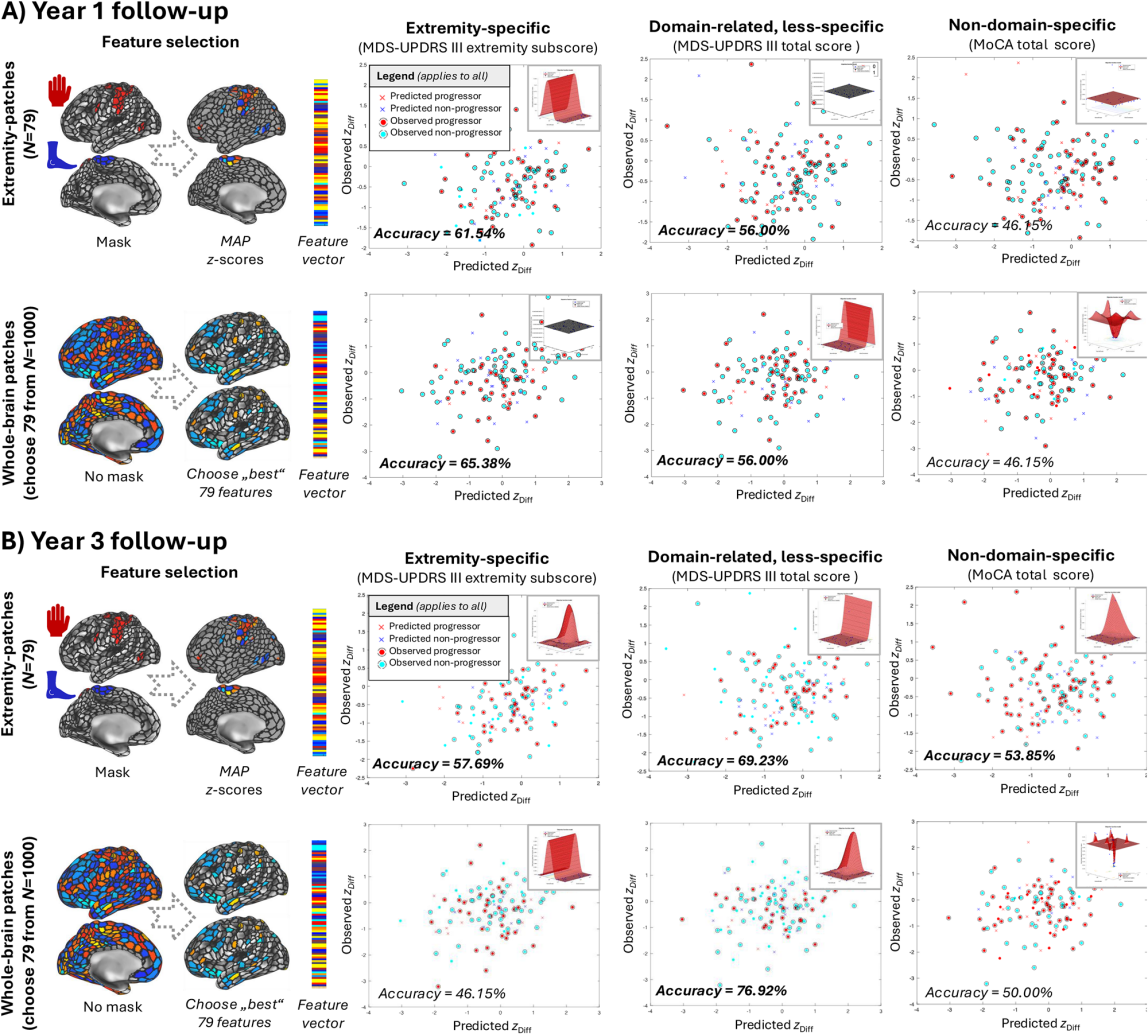
Classification results. We compared the performance of two support vector machine (SVM) models in predicting 1‐year (Y1, A)/3‐year (Y3, B) clinical scores using standardized baseline (BL) information from either an extremity‐specific restricted thin‐patch fraction (TPF), only considering domain‐specific patches corresponding to “extremity function” (upper row, cf. Methods section and Figure 1 for further details) and unrestricted whole‐brain TPF (lower row). Feature selection in the whole‐brain SVM model was set to *N* = 79 patches, that is, features, to match the number of the extremity‐specific SVM, for which no further feature selection was performed. Columns correspond to different clinical functions, namely the extremity‐specific motor function (a subscore from the MDS‐UPDRS III, cf. methods section), domain‐related but less‐specific general motor function (MDS‐UPDRS III total scores), and nondomain‐specific cognitive function (MoCA total scores). As input data, we used *z*‐standardized cortical thickness (CTh) values from each patch following the Mosaic Approach (MAP, cf. Methods section). Red crosses indicate predicted progressors, red circles observed progressors, and vice versa for nonprogressors and blue symbols. The accuracy of each model is indicated in the lower left corner. The insert in the upper right corner represents the best‐fitting hyperplane. MAP = Mosaic Approach, MoCA = Montreal Cognitive Assessment, ROI = region of interest, TPF = thin‐patch fraction, MDS‐UPDRS = Unified Parkinson's Disease Rating Scale, modified revision sponsored by the Movement Disorder Society.

In our final analysis, we contrasted the performance of two SVMs in predicting future (Y1/Y3) clinical scores, using baseline, MAP‐standardized CTh data. The first SVM used information from the domain‐specific TPF, only considering the *N* = 79 patches corresponding to the extremity function (cf. Methods section and above), while the second SVM used information from all *N =* 1000 unrestricted whole‐brain patches but setting the number of features‐to‐select to *N* = 79 to enhance comparability to the first model. Figure 4 shows the results: The accuracy in predicting Y1 progressors versus nonprogressors (Figure 4A) for extremity‐specific motor function was above chance for both domain‐specific and unrestricted whole‐brain TPF (accuracy extremity‐specific TPF = 61.54%, accuracy unrestricted whole‐brain TPF = 65.38%). Likewise, the accuracy was above chance in predicting Y1 progressors versus nonprogressors for general motor function, however lower as compared with extremity‐specific function (accuracy extremity‐specific TPF = 56.00%, accuracy unrestricted whole‐brain TPF = 56.00%). Domain‐unspecific cognitive progressors at Y1 could not be classified above chance using extremity‐specific or unrestricted whole‐brain TPF at baseline (accuracy extremity‐specific TPF = 46.15%, accuracy unrestricted whole‐brain TPF = 46.15%).

The accuracy in predicting Y3 progressors versus nonprogressors (Figure 4B) for the extremity‐specific motor function was above chance for the domain‐specific TPF (accuracy = 57.69%), but not for the unrestricted whole‐brain TPF (accuracy 46.15%). For general motor function, accuracy was above chance in predicting Y3 progressors versus nonprogressors was above chance for both the domain‐specific and the unrestricted TPFs; however, much higher for the unrestricted TPF (accuracy extremity‐specific TPF = 69.23%, accuracy unrestricted whole‐brain TPF = 76.92%). Domain‐unspecific cognitive progressors at Y3 could barely be classified above chance using extremity‐specific or unrestricted whole‐brain TPF at baseline (accuracy extremity‐specific TPF = 53.85%, accuracy unrestricted whole‐brain TPF = 50.00%).

## 4 Conclusion

The present study evaluated the utility of a normative modeling framework for domain‐specific prediction of clinical disability in Parkinson's Disease. Specifically, we investigated the recently proposed Mosaic Approach (MAP), which assesses structural cortical disease burden for a high‐resolution parcellation of the human cortex for individuals with respect to a normative control cohort. We hypothesized that restricting the traditional MAP approach—which covers the entire cortex—to regions functionally associated with the clinical symptom of interest would enhance the explanatory power of corresponding domain‐specific symptom severity, and could even predict progression over the course of 1 and 3 years. Our results supported these hypotheses: we restricted the MAP framework to regions topographically associated with motor function of the extremities and found increased explanatory power of this restricted approach compared with the unrestricted MAP approach for extremity‐specific motor scores. Moreover, the restricted MAP approach still yielded significant explanatory power for the domain‐related but less specific general motor function, however, less as compared with the extremity‐specific MAP approach. In line with this, and in reverse, the unrestricted MAP approach yielded higher explanatory power for the domain‐related but less specific general motor function compared with the extremity‐specific motor function. In an additional control condition, we investigated nondomain‐specific cognitive function, with which neither restricted nor unrestricted approaches were significantly associated. In a second analysis, we probed the predictive power of the restricted MAP approach in classifying progressors from nonprogressors 1‐ and 3 years later using baseline data. Indeed, at the 1‐year follow‐up, the accuracy of this classification was enhanced for predicting domain‐specific progressors compared with nondomain‐specific progressors. However, also the unrestricted approach yielded similar levels of accuracy, suggesting that along the PD disease course, also domain‐unrelated cortical regions become relevant. We found similar results for the 3‐year follow‐up. However, we observed a tendency for enhanced discriminatory power of our approach since the accuracy for predicting domain‐specific progressors was higher versus the 1‐year follow‐up, while the accuracy for predicting nondomain‐specific progressors was below chance. This might reflect a higher cortical disease burden with ongoing disease activity in PD (Nürnberger et al. 2017). In line with this, the accuracy for predicting nondomain‐specific progressors using the unrestricted MAP approach at the 3‐year follow‐up was considerably higher versus at the 1‐year follow‐up. This might also explain why at the 3‐year follow‐up, we observed increased accuracy for predicting nondomain‐specific progressors as compared with the 1‐year follow‐up with the restricted MAP approach, potentially mimicking widespread brain pathology in PD as the disease unfolds (Yang, Burciu, and Vaillancourt 2018).

The observation that as PD progresses, various cortical regions begin to associate with a given function of interest might reflect ongoing neuroplastic changes. Neural plasticity describes the ability of the central nervous system (CNS) to adapt to environmental changes including CNS lesions (Sharma, Classen, and Cohen 2013). For example, in patients with MS—a disorder characterized by such lesions (Reich, Lucchinetti, and Calabresi 2018)—cortical areas are found to control movements not typically found in healthy controls (Pantano et al. 2002; Reddy et al. 2002; Tahedl et al. 2022a). This is observed even in the absence of clinical disability, such that it is believed that in response to MS lesions, neuronal networks reorganize and recruit additional cortical regions to maintain a domain‐specific function (Acharya et al. 2012; Zotey et al. 2023). This could also explain why we observed high accuracy of domain‐unrelated cortical regions to predict future disease course: in response to PD‐related atrophy, neuronal reorganization might occur, such that baseline structural assessment of those regions is a predictor of the clinical course since they might reflect the level of brain reserve. Indeed, evidence from neuroimaging studies has suggested that brain reserve might be protective of developing severe symptoms in PD (Hindle, Martyr, and Clare 2014; Hoenig et al. 2023), which is further supported by our results.

However, we also provided evidence that when assessing a domain‐specific function of interest in PD, restricting the cortex to “classically”‐associated, domain‐specific cortical regions enhance the explanatory power of that function and can even facilitate prediction. In the terminology of machine learning, this can be understood as “feature selection”. When developing ML‐based classifiers, the data, that is, the “features”, on which to train the algorithm is crucial for the performance of the classifier (Cai et al. 2018): the more the features represent the function of interest, the higher the ultimate accuracy of the algorithm (Chu et al. 2012). Especially for neuroimaging‐based ML classifiers an informed selection of features is crucial since otherwise each brain voxel of the MRI data will be treated as a feature, easily resulting in hundreds of thousands of features. Such expansive dimensionality not only can produce computational problems but also requires increasing numbers of training data sets (Hua et al. 2005), which are however limited in neuroimaging analyses. Therefore, reducing the number of features to a minimal amount with maximum quality is therefore desirable, especially for smaller studies. We have shown that the MAP framework offers a strategy for such delicate feature selection: MAP outputs measure quantifying individual cortical disease burden with respect to a population‐expected norm for a high‐resolution parcellation of the human cortex. This high resolution allows for the straightforward identification of parcels corresponding to a domain‐specific function of interest, which allows for the computationally efficient development of ML classifiers.

### 4.1 Limitations

Our study is limited for various reasons: First, we only tested for extremity‐specific motor function over a relatively short follow‐up period of 1 year, and the structural baseline MRI data we analyzed was from newly diagnosed PD patients, such that we claim no generalizability. Especially, generalizability to the cognitive domain might be problematic based on our current analysis for several reasons: (1) the topography of fMRI maps reflecting cognitive subdomains is inconsistently described throughout the literature (Uddin, Yeo, and Spreng 2019; Uddin et al. 2023; Winston 2018), but our present analysis was investigating a topographically very clearly and specifically‐defined region‐ and associated function‐of‐interest (i.e., extremity‐specific motor cortex and associated function). (2) TPF is a gray matter metric. However, studies have shown that for memory performance, especially in older adults, gray matter metrics yield little or no predictive power, whereas task‐based fMRI contrasts and/or resting‐state fMRI maps do (Soch et al. 2022). In that study, the authors hypothesized that memory performance might be more closely related to differences in recruitment efficiency of memory networks than to brain structure, which is why our current analysis might not be ideal for the assessment of cognitive (sub‐)functions, which will, however, need to be assessed dedicatedly in future work.

Second, the dichotomization approach we used for defining progressors from nonprogressors might be seen critically, since a small change in the MDS‐UPDRS over 1/3 years might reflect interrater variability and/or day‐to‐day variation in some instances. However, in spite of this shortcoming, we believe it was the most reasonable choice for our study design, for two reasons: (1) A 2017 study investigating the PPMI dataset (Holden et al. 2018) found that the average annual increase of MDS‐UPDRS III was 2.4 points, such that a higher cut‐off, for example, a 5‐ or even 10‐point‐change, would leave hardly any patients left for the prediction analysis. However, there is also good evidence that the MDS‐UPDRS demonstrates good reliability, validity, and sensitivity to changes (van Hilten et al. 1994; Martínez‐Martín et al. 1994; Rabey et al. 1997; Ramaker et al. 2002; Stebbins et al. 1999). The variability in annual MDS‐UPDRS III changes is believed to be mostly due to the lack of consideration of disease duration, potentially also disease severity, and inconsistent MDS‐UPDRS administration (Holden et al. 2018). In our model, we therefore included symptom duration as a confounding variable. Moreover, the PPMI project puts much emphasis on standardized clinical assessment and training of the involved clinical personnel (Marek et al. 2011), such that we are confident that most of the annual changes reported in our study are due to real clinical worsening.

Third, the prognostic performance of our present analysis is currently limited, since both the adjusted *R^2^* values are relatively small and the prediction accuracies are only slightly above chance, especially for the 1‐year follow‐up. However, since we were able to replicate our prediction analysis for a further timepoint (i.e., the 3‐year follow‐up)—where the accuracies further increased—we are confident that our method holds true potential for making such delicate classifications. Nevertheless, in its current form, our present results should be taken as a proof‐of‐principle, needing further replication in future work.

We would also like to note that due to data restriction reasons, we could not account for the potential confounding effects of different MRI manufacturers and different scanning sites, which might affect cortical thickness calculations using FreeSurfer (Hedges et al. 2022; Knussmann et al. 2022).

Moreover, given cortical atrophy reflects only one aspect of cerebral disease manifestation in PD—next to various others including basal ganglia volume and neuromelanin content of the substantia nigra (Bloem, Okun, and Klein 2021)—a biomarker derived from this approach is likewise limited. Nevertheless, our results support the notion of MAP as a promising strategy for aiding individual domain‐specific clinical progression in PD. When combined with other cerebral biomarkers, MAP might be an invaluable contribution to personalized PD patient care, which may be further explored in future studies.

## Author Contributions

**Marlene Tahedl**: conceptualization, formal analysis, investigation, methodology, software, visualization, writing–original draft. **Ulrich Bogdahn**: conceptualization, supervision, writing–review and editing. **Bernadette Wimmer**: conceptualization, writing–review and editing. **Dennis Martin Hedderich**: project administration, resources, writing–review and editing. **Jan Kirschke**: project administration, resources, writing–review and editing. **Claus Zimmer**: project administration, resources, writing–review and editing. **Benedikt Wiestler**: conceptualization, investigation, project administration, resources, supervision, writing–review and editing.

## Ethics Statement

The PPMI study has been approved by the respective institutional review boards of all participating sites, and written informed consent were obtained from all participants.

## Conflicts of Interest

J.S.K. has received speaker fees from Novartis. D.M.H. has received speaker fees from Eisai GmbH. The other authors declare no conflicts of interest. All disclosures are not relevant to the present study. M.T. is funded by a Walter‐Benjamin stipend from the Deutsche Forschungsgemeinschaft (DFG, TA 1902/1‐1).

### Peer Review

The peer review history for this article is available at https://publons.com/publon/10.1002/brb3.70289.

## PPMI Study Group

https://www.ppmi‐info.org/sites/default/files/docs/ppmi‐publication‐policy.pdf

## Data Availability

The raw data (neuroimaging and clinical) used in this project were obtained from the PPMI, an open‐access dataset, open to researchers upon reasonable request. There are four tiers of the PPMI data; the data used herein fall under the category *Tier 1* (“publicly available and refreshed daily”). The processed data and statistics may be obtained from the corresponding author upon request.

## References

[brb370289-bib-0001] Acharya, S. , S. Shukla , S. N. Mahajan , and S. K. Diwan . 2012. “Localizationism to Neuroplasticity—The Evolution of Metaphysical Neuroscience.” Journal of Association of Physicians of India 60: 38–46. 23547412

[brb370289-bib-0002] Bethlehem, R. A. I. , J. Seidlitz , S. R. White , et al. 2022. “Brain Charts for the Human Lifespan.” Nature 604: 525–533. 35388223 10.1038/s41586-022-04554-y PMC9021021

[brb370289-bib-0003] Bloem, B. R. , M. S. Okun , and C. Klein . 2021. “Parkinson's Disease.” The Lancet 397: 2284–2303. 10.1016/S0140-6736(21)00218-X 33848468

[brb370289-bib-0004] Buckner, R. L. , F. M. Krienen , A. Castellanos , J. C. Diaz , and B. T. T. Yeo . 2011. “The Organization of the human Cerebellum Estimated by Intrinsic Functional Connectivity.” Journal of Neurophysiology 106: 2322–2345. https://www.physiology.org/doi/10.1152/jn.00339.2011. 21795627 10.1152/jn.00339.2011 PMC3214121

[brb370289-bib-0005] Cai, J. , J. Luo , S. Wang , and S. Yang . 2018. “Feature Selection in Machine Learning: A New Perspective.” Neurocomputing 300: 70–79.

[brb370289-bib-0006] Chu, C. , A.‐L. Hsu , K.‐H. Chou , P. Bandettini , and C. Lin . 2012. “Does Feature Selection Improve Classification Accuracy? Impact of Sample Size and Feature Selection on Classification Using Anatomical Magnetic Resonance Images.” Neuroimage 60: 59–70. 22166797 10.1016/j.neuroimage.2011.11.066

[brb370289-bib-0007] Dadu, A. , V. Satone , R. Kaur , et al. 2022. “Identification and Prediction of Parkinson's Disease Subtypes and Progression Using Machine Learning in Two Cohorts.” NPJ Parkinson's Disease 8: 172. 10.1038/s41531-022-00439-z PMC9758217 36526647

[brb370289-bib-0008] Del Tredici, K. , and H. Braak . 2012. “Spinal Cord Lesions in Sporadic Parkinson's Disease.” Acta Neuropathologica 124: 643–664. 22926675 10.1007/s00401-012-1028-y

[brb370289-bib-0009] Del Tredici, K. , and H. Braak . 2016. “Review: Sporadic Parkinson's Disease: Development and Distribution of α‐Synuclein Pathology.” Neuropathology and Applied Neurobiology 42: 33–50. 26662475 10.1111/nan.12298

[brb370289-bib-0010] Deng, X. , Z. Liu , Q. Kang , L. Lu , Y. Zhu , and R. Xu . 2021. “Cortical Structural Connectivity Alterations and Potential Pathogenesis in Mid‐Stage Sporadic Parkinson's Disease.” Frontiers in Aging Neuroscience 13: 650371. 34135748 10.3389/fnagi.2021.650371 PMC8200851

[brb370289-bib-0011] Di Biase, M. A. , Y. E. Tian , R. A. I. Bethlehem , et al. 2023. “Mapping Human Brain Charts Cross‐sectionally and Longitudinally.” PNAS 120, no. 20:e2216798120. 37155868 10.1073/pnas.2216798120 PMC10193972

[brb370289-bib-0012] Dickie, E. W. , A. Anticevic , D. E. Smith , et al. 2019. “Ciftify: A Framework for Surface‐based Analysis of Legacy MR Acquisitions.” Neuroimage 197: 818–826. https://linkinghub.elsevier.com/retrieve/pii/S1053811919303714. 31091476 10.1016/j.neuroimage.2019.04.078 PMC6675413

[brb370289-bib-0013] Domínguez‐Fernández, C. , J. Egiguren‐Ortiz , J. Razquin , et al. 2023. “Review of Technological Challenges in Personalised Medicine and Early Diagnosis of Neurodegenerative Disorders.” International Journal of Molecular Sciences 24, no. 4: 3321. 36834733 10.3390/ijms24043321 PMC9968142

[brb370289-bib-0014] Ebadi, A. , J. L. Dalboni da Rocha , D. B. Nagaraju , et al. 2017. “Ensemble Classification of Alzheimer's Disease and Mild Cognitive Impairment Based on Complex Graph Measures From Diffusion Tensor Images.” Frontiers in Neuroscience 11: 56. 28293162 10.3389/fnins.2017.00056 PMC5329061

[brb370289-bib-0015] Fischl, B. 2012. “FreeSurfer.” Neuroimage 62: 774–781. https://linkinghub.elsevier.com/retrieve/pii/S1053811912000389. 22248573 10.1016/j.neuroimage.2012.01.021 PMC3685476

[brb370289-bib-0016] Foffani, G. , and J. A. Obeso . 2018. “A Cortical Pathogenic Theory of Parkinson's Disease.” Neuron 99: 1116–1128. 30236282 10.1016/j.neuron.2018.07.028

[brb370289-bib-0017] Goetz, C. G. , B. C. Tilley , S. R. Shaftman , et al. 2008. “Movement Disorder Society‐Sponsored Revision of the Unified Parkinson's Disease Rating Scale (MDS‐UPDRS): Scale Presentation and Clinimetric Testing Results.” Movement Disorders 23: 2129–2170. 19025984 10.1002/mds.22340

[brb370289-bib-0018] Greenland, J. C. , C. H. Williams‐Gray , and R. A. Barker . 2019. “The Clinical Heterogeneity of Parkinson's Disease and Its Therapeutic Implications.” European Journal of Neuroscience 49: 328–338. 30059179 10.1111/ejn.14094

[brb370289-bib-0019] He, P. , Y. Gao , L. Shi , et al. 2023. “Motor Progression Phenotypes in Early‐stage Parkinson's Disease: a Clinical Prediction Model and the Role of Glymphatic System Imaging Biomarkers.” Neuroscience Letters 814: 137435. 37562710 10.1016/j.neulet.2023.137435

[brb370289-bib-0020] Hedges, E. P. , M. Dimitrov , U. Zahid , et al. 2022. “Reliability of Structural MRI Measurements: The Effects of Scan Session, Head Tilt, Inter‐Scan Interval, Acquisition Sequence, FreeSurfer Version and Processing Stream.” Neuroimage 246: 118751. 34848299 10.1016/j.neuroimage.2021.118751 PMC8784825

[brb370289-bib-0021] Hindle, J. V. , A. Martyr , and L. Clare . 2014. “Cognitive Reserve in Parkinson's disease: A Systematic Review and Meta‐analysis.” Parkinsonism & Related Disorders 20: 1–7. 24034887 10.1016/j.parkreldis.2013.08.010

[brb370289-bib-0022] Hoehn, M. M. , and M. D. Yahr . 1998. “Parkinsonism: Onset, Progression, and Mortality. 1967.” Neurology 50: 318 and 16 pages following. 9484345 10.1212/wnl.50.2.318

[brb370289-bib-0023] Hoenig, M. C. , V. Dzialas , A. Drzezga , and T. van Eimeren . 2023. “The Concept of Motor Reserve in Parkinson's Disease: New Wine in Old Bottles?” Movement Disorders 38: 16–20. 36345092 10.1002/mds.29266

[brb370289-bib-0024] Holden, S. K. , T. Finseth , S. H. Sillau , and B. D. Berman . 2018. “Progression of MDS‐UPDRS Scores Over Five Years in De Novo Parkinson Disease From the Parkinson's Progression Markers Initiative Cohort.” Movement Disorders Clinical Practice 5: 47–53. 29662921 10.1002/mdc3.12553 PMC5898442

[brb370289-bib-0025] Horsager, J. , K. B. Andersen , K. Knudsen , et al. 2020. “Brain‐First Versus Body‐first Parkinson's Disease: A Multimodal Imaging Case‐Control Study.” Brain 143: 3077–3088. 32830221 10.1093/brain/awaa238

[brb370289-bib-0026] Hua, J. , Z. Xiong , J. Lowey , E. Suh , and E. R. Dougherty . 2005. “Optimal Number of Features as a Function of Sample Size for Various Classification Rules.” Bioinformatics 21: 1509–1515. 15572470 10.1093/bioinformatics/bti171

[brb370289-bib-0027] Johansson, M. E. , I. Toni , R. P. C. Kessels , B. R. Bloem , and R. C. Helmich . 2024. “Clinical Severity in Parkinson's Disease Is Determined by Decline in Cortical Compensation.” Brain 147: 871–886. 37757883 10.1093/brain/awad325 PMC10907095

[brb370289-bib-0028] Kalia, L. V. , and A. E. Lang . 2015. “Parkinson's Disease.” Lancet 386: 896–912. 25904081 10.1016/S0140-6736(14)61393-3

[brb370289-bib-0029] Knussmann, G. N. , J. S. Anderson , M. B. D. Prigge , et al. 2022. “Test‐Retest Reliability of FreeSurfer‐derived Volume, Area and Cortical Thickness From MPRAGE and MP2RAGE Brain MRI Images.” *Neuroimage Reports* 2. 10.1016/j.ynirp.2022.100086 PMC9409374 36032692

[brb370289-bib-0030] Leal, D. A. B. , C. M. V. Dias , R. P. Ramos , and I. Brys . 2023. “Prediction of Dyskinesia in Parkinson's Disease Patients Using Machine Learning Algorithms.” Scientific Reports 13: 22426. 38104147 10.1038/s41598-023-49617-w PMC10725420

[brb370289-bib-0031] Marek, K. , D. Jennings , S. Lasch , et al. 2011. “The Parkinson Progression Marker Initiative (PPMI).” Progress in Neurobiology 95: 629–635. 21930184 10.1016/j.pneurobio.2011.09.005 PMC9014725

[brb370289-bib-0032] Martínez‐Martín, P. , A. Gil‐Nagel , L. M. Gracia , J. B. Gómez , J. Martínez‐Sarriés , and F. Bermejo . 1994. “Unified Parkinson's Disease Rating Scale Characteristics and Structure.” Movement Disorders 9: 76–83. 8139608 10.1002/mds.870090112

[brb370289-bib-0033] McKenna, M. C. , M. Tahedl , J. Lope , et al. 2022. “Mapping Cortical Disease‐Burden at Individual‐Level in Frontotemporal Dementia: Implications for Clinical Care and Pharmacological Trials.” Brain Imaging and Behavior 16: 1196–1207. http://www.ncbi.nlm.nih.gov/pubmed/34882275. 34882275 10.1007/s11682-021-00523-7 PMC9107414

[brb370289-bib-0034] Mishima, T. , S. Fujioka , T. Morishita , T. Inoue , and Y. Tsuboi . 2021. “Personalized Medicine in Parkinson's Disease: New Options for Advanced Treatments.” Journal of Personalized Medicine 11: 650. 34357117 10.3390/jpm11070650 PMC8303729

[brb370289-bib-0035] Nasreddine, Z. S. , N. A. Phillips , V. Bédirian , et al. 2014. Montreal Cognitive Assessment. PsycTESTS Dataset.

[brb370289-bib-0036] Nenning, K.‐H. , and G. Langs . 2022. “Machine Learning in Neuroimaging: From Research to Clinical Practice.” Die Radiologie 62: 1–10. 36044070 10.1007/s00117-022-01051-1 PMC9732070

[brb370289-bib-0037] Nürnberger, L. , R.‐M. Gracien , P. Hok , et al. 2017. “Longitudinal Changes of Cortical Microstructure in Parkinson's Disease Assessed With T1 Relaxometry.” NeuroImage. Clinical 13: 405–414. 28116233 10.1016/j.nicl.2016.12.025 PMC5226811

[brb370289-bib-0038] Pantano, P. , G. D. Iannetti , F. Caramia , et al. 2002. “Cortical Motor Reorganization After a Single Clinical Attack of Multiple Sclerosis.” Brain 125: 1607–1615. http://www.ncbi.nlm.nih.gov/pubmed/12077009. 12077009 10.1093/brain/awf164

[brb370289-bib-0039] Rabey, J. M. , H. Bass , U. Bonuccelli , et al. 1997. “Evaluation of the Short Parkinson's Evaluation Scale: A New Friendly Scale for the Evaluation of Parkinson's Disease in Clinical Drug Trials.” Clinical Neuropharmacology 20: 322–337. 9260730

[brb370289-bib-0040] Ramaker, C. , J. Marinus , A. M. Stiggelbout , and B. J. van Hilten . 2002. “Systematic Evaluation of Rating Scales for Impairment and Disability in Parkinson's Disease.” Movement Disorders 17: 867–876. 12360535 10.1002/mds.10248

[brb370289-bib-0041] Reddy, H. , S. Narayanan , M. Woolrich , et al. 2002. “Functional Brain Reorganization for Hand Movement in Patients With Multiple Sclerosis: Defining Distinct Effects of Injury and Disability.” Brain 125: 2646–2657. http://www.ncbi.nlm.nih.gov/pubmed/12429592. 12429592 10.1093/brain/awf283

[brb370289-bib-0042] Reich, D. S. , C. F. Lucchinetti , and P. A. Calabresi . 2018. “Multiple Sclerosis.” New England Journal of Medicine 378: 169–180. http://www.ncbi.nlm.nih.gov/pubmed/29320652. 29320652 10.1056/NEJMra1401483 PMC6942519

[brb370289-bib-0043] RStudio Team . 2022. RStudio: Integrated Development Environment for R. Boston, MA. http://www.rstudio.com/.

[brb370289-bib-0044] Rubinov, M. , and O. Sporns . 2010. “Complex Network Measures of Brain Connectivity: Uses and Interpretations.” Neuroimage 52: 1059–1069. http://www.ncbi.nlm.nih.gov/pubmed/19819337. 19819337 10.1016/j.neuroimage.2009.10.003

[brb370289-bib-0045] Schaefer, A. , R. Kong , E. M. Gordon , et al. 2018. “Local‐Global Parcellation of the Human Cerebral Cortex From Intrinsic Functional Connectivity MRI.” Cerebral Cortex 28: 3095–3114. https://academic.oup.com/cercor/article/28/9/3095/3978804. 28981612 10.1093/cercor/bhx179 PMC6095216

[brb370289-bib-0046] Shafto, M. , L. K. Tyler , M. Dixon , et al. 2014. “The Cambridge Centre for Ageing and Neuroscience (Cam‐CAN) Study Protocol: a Cross‐sectional, Lifespan, Multidisciplinary Examination of Healthy Cognitive Ageing.” BMC Neurology [Electronic Resource] 14: 204. http://www.pubmedcentral.nih.gov/articlerender.fcgi?artid=4219118&tool=pmcentrez&rendertype=abstract. 25412575 10.1186/s12883-014-0204-1 PMC4219118

[brb370289-bib-0047] Sharma, N. , J. Classen , and L. G. Cohen . 2013. “Neural Plasticity and Its Contribution to Functional Recovery.” Handbook of Clinical Neurology 110: 3–12. http://www.ncbi.nlm.nih.gov/pubmed/23312626. 23312626 10.1016/B978-0-444-52901-5.00001-0 PMC4880010

[brb370289-bib-0048] Shimron, E. , and O. Perlman . 2023. “AI in MRI: Computational Frameworks for a Faster, Optimized, and Automated Imaging Workflow.” Bioengineering 10: 492. 37106679 10.3390/bioengineering10040492 PMC10135995

[brb370289-bib-0049] Soch, J. , A. Richter , J. M. Kizilirmak , et al. 2022. “Structural and Functional MRI Data Differentially Predict Chronological Age and Behavioral Memory Performance.” *Eneuro* 9. 10.1523/ENEURO.0212-22.2022 PMC9665883 36376083

[brb370289-bib-0050] Stebbins, G. T. , C. G. Goetz , A. E. Lang , and E. Cubo . 1999. “Factor Analysis of the Motor Section of the Unified Parkinson's Disease Rating Scale During the Off‐State.” Movement Disorders 14: 585–589. 10435494 10.1002/1531-8257(199907)14:4<585::aid-mds1006>3.0.co;2-3

[brb370289-bib-0051] Tahedl, M. 2020. “Towards Individualized Cortical Thickness Assessment for Clinical Routine.” Journal of Translational Medicine 18: 1–12. 10.1186/s12967-020-02317-9. 32245485 PMC7118882

[brb370289-bib-0052] Tahedl, M. , R. H. Chipika , J. Lope , S. Li Hi Shing , O. Hardiman , and P. Bede . 2021. “Cortical Progression Patterns in Individual ALS Patients Across Multiple Timepoints: a Mosaic‐based Approach for Clinical Use.” Journal of Neurology 268: 1913–1926. https://link.springer.com/10.1007/s00415‐020‐10368‐7. 33399966 10.1007/s00415-020-10368-7

[brb370289-bib-0053] Tahedl, M. , S. M. Levine , R. Weissert , et al. 2022a. “Early Remission in Multiple Sclerosis Is Linked to Altered Coherence of the Cerebellar Network.” Journal of Translational Medicine 20: 488. https://translational‐medicine.biomedcentral.com/articles/10.1186/s12967‐022‐03576‐4. 36303221 10.1186/s12967-022-03576-4 PMC9615296

[brb370289-bib-0054] Tahedl, M. , S. Li Hi Shing , E. Finegan , et al. 2022b. “Propagation Patterns in Motor Neuron Diseases: Individual and Phenotype‐associated Disease‐Burden Trajectories Across the UMN‐LMN Spectrum of MNDs.” Neurobiology of Aging 109: 78–87. https://linkinghub.elsevier.com/retrieve/pii/S0197458021003067. 34656922 10.1016/j.neurobiolaging.2021.04.031

[brb370289-bib-0055] Tahedl, M. , and J. V. Schwarzbach . 2023. “An Automated Pipeline for Obtaining Labeled ICA‐templates Corresponding to Functional Brain Systems.” Human Brain Mapping 44: 5202–5211. 37516917 10.1002/hbm.26435 PMC10543103

[brb370289-bib-0056] Tahedl, M. , T. Wiltgen , C. C. Voon , et al. 2023. “Benefits of a Mosaic Approach for Assessing Cortical Atrophy in Individual Multiple Sclerosis Patients.” Brain and Behavior 13: e3327. 37961043 10.1002/brb3.3327 PMC10726853

[brb370289-bib-0057] Tahedl, M. , T. Wiltgen , C. C. Voon , et al. 2024. “Cortical Thin Patch Fraction Reflects Disease Burden in MS: The Mosaic Approach.” American Journal of Neuroradiology 45: 82–89. 10.3174/ajnr.A8064 PMC10756581 38164526

[brb370289-bib-0058] Uddin, L. Q. , R. F. Betzel , J. R. Cohen , et al. 2023. “Controversies and Progress on Standardization of Large‐Scale Brain Network Nomenclature.” Network Neuroscience 7: 864–905. 37781138 10.1162/netn_a_00323 PMC10473266

[brb370289-bib-0059] Uddin, L. Q. , B. T. T. Yeo , and R. N. Spreng . 2019. “Towards a Universal Taxonomy of Macro‐Scale Functional Human Brain Networks.” Brain Topography 32: 926–942. 31707621 10.1007/s10548-019-00744-6 PMC7325607

[brb370289-bib-0060] Van Essen, D. C. , S. M. Smith , D. M. Barch , et al. WU‐Minn HCP Consortium . 2013. “The WU‐Minn Human Connectome Project: An Overview.” Neuroimage 80: 62–79. http://www.ncbi.nlm.nih.gov/pubmed/23684880. 23684880 10.1016/j.neuroimage.2013.05.041 PMC3724347

[brb370289-bib-0061] van Hilten, J. J. , A. D. van der Zwan , A. H. Zwinderman , and R. A. C. Roos . 1994. “Rating Impairment and Disability in Parkinson's Disease: Evaluation of the Unified Parkinson's Disease Rating Scale.” Movement Disorders 9: 84–88. 8139609 10.1002/mds.870090113

[brb370289-bib-0062] Vapnik, V. , and A. Lerner . 1963. “Pattern Recognition Using Generalized Portrait Method.” Automation and Remote Control 24: 774–780.

[brb370289-bib-0063] Winston, J. E. 2018. “Twenty‐First Century Biological Nomenclature—The Enduring Power of Names.” Integrative and Comparative Biology 58, no. 6: 1122–1131. 30113637 10.1093/icb/icy060

[brb370289-bib-0064] Yang, J. , R. G. Burciu , and D. E. Vaillancourt . 2018. “Longitudinal Progression Markers of Parkinson's Disease: Current View on Structural Imaging.” Current Neurology and Neuroscience Reports 18: 83. 30280267 10.1007/s11910-018-0894-7 PMC8650526

[brb370289-bib-0065] Yarkoni, T. , R. A. Poldrack , T. E. Nichols , D. C. Van Essen , and T. D. Wager . 2011. “Large‐Scale Automated Synthesis of Human Functional Neuroimaging Data.” Nature Methods 8: 665–670. 21706013 10.1038/nmeth.1635 PMC3146590

[brb370289-bib-0066] Yeo, B. T. T. , F. M. Krienen , J. Sepulcre , et al. 2011. “The Organization of the Human Cerebral Cortex Estimated by Intrinsic Functional Connectivity.” Journal of Neurophysiology 106: 1125–1165. 21653723 10.1152/jn.00338.2011 PMC3174820

[brb370289-bib-0067] Zotey, V. , A. Andhale , T. Shegekar , and A. Juganavar . 2023. “Adaptive Neuroplasticity in Brain Injury Recovery: Strategies and Insights.” Cureus 15: e45873. 37885532 10.7759/cureus.45873 PMC10598326

